# ProtNote: a multimodal method for protein–function annotation

**DOI:** 10.1093/bioinformatics/btaf170

**Published:** 2025-04-15

**Authors:** Samir Char, Nathaniel Corley, Sarah Alamdari, Kevin K Yang, Ava P Amini

**Affiliations:** Microsoft Cloud & AI, Microsoft, Redmond, WA 98052, United States; Microsoft Cloud & AI, Health & Life Sciences, Microsoft, Redmond, WA 98052, United States; Microsoft Research, Microsoft, 1 Memorial Dr, Cambridge, MA 02142, United States; Microsoft Research, Microsoft, 1 Memorial Dr, Cambridge, MA 02142, United States; Microsoft Research, Microsoft, 1 Memorial Dr, Cambridge, MA 02142, United States

## Abstract

**Motivation:**

Understanding the protein sequence–function relationship is essential for advancing protein biology and engineering. However, <1% of known protein sequences have human-verified functions. While deep-learning methods have demonstrated promise for protein–function prediction, current models are limited to predicting only those functions on which they were trained.

**Results:**

Here, we introduce ProtNote, a multimodal deep-learning model that leverages free-form text to enable both supervised and zero-shot protein–function prediction. ProtNote not only maintains near state-of-the-art performance for annotations in its training set but also generalizes to unseen and novel functions in zero-shot test settings. ProtNote demonstrates superior performance in the prediction of novel Gene Ontology annotations and Enzyme Commission numbers compared to baseline models by capturing nuanced sequence–function relationships that unlock a range of biological use cases inaccessible to prior models. We envision that ProtNote will enhance protein–function discovery by enabling scientists to use free text inputs without restriction to predefined labels—a necessary capability for navigating the dynamic landscape of protein biology.

**Availability and Implementation:**

The code is available on GitHub: https://github.com/microsoft/protnote; model weights, datasets, and evaluation metrics are provided via Zenodo: https://zenodo.org/records/13897920.

## 1 Introduction

**Figure 1.**
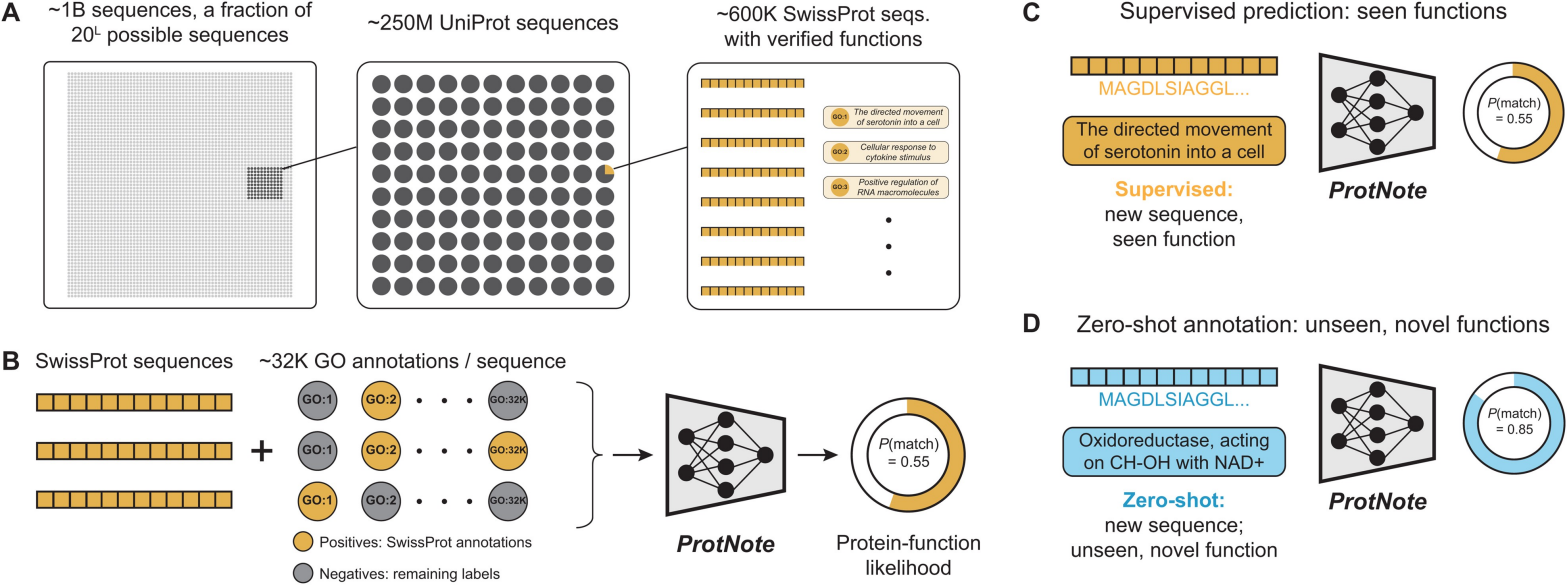
Protein–function annotation with ProtNote. (A) A small fraction of all possible protein sequences are recorded in the UniProt database (The UniProt Consortium 2022). An even smaller portion, available in SwissProt (Boutet *et al.* 2007), has human-verified functions. Text descriptions of function are assigned Gene Ontology term labels. (B) For a given sequence from SwissProt, positive labels are applicable true SwissProt annotations; negative terms are the labels that do not apply to the sequence and are pulled from the Gene Ontology database (Ashburner *et al.* 2000; Gene Ontology Consortium *et al.* 2023). ProtNote is trained on amino acid sequences and the textual descriptions from the positive and negative GO terms and outputs a predicted protein–function likelihood for each input pair. (C and D) At inference, ProtNote can be deployed in both supervised (C) and zero-shot (D) settings. ProtNote takes unseen protein–function pairs as inputs and outputs the probability that the proteins perform the functions they were paired with, in both supervised prediction over seen functions (C) and zero-shot annotation over unseen, novel functions (D).

Proteins, the building blocks of cellular life, exhibit astonishing functional diversity. Indeed, in domains as varied as medicine (Leader *et al.* 2008, Dimitrov 2012), agriculture (Mann *et al.* 2023), and the food industry (Raveendran *et al.* 2018), scientists continue to discover novel and valuable applications for proteins. Before applying proteins to downstream tasks, practitioners must first understand their functions. However, high-fidelity function annotations are sparse, with fewer than 1% of the sequence entries in the UniProt database (The UniProt Consortium 2022) containing human-verified functions (Fig. 1A). Developing tools to predict protein–function from sequence automatically is paramount not only to inform our biochemical understanding of proteins but also to accelerate applications to an expanding set of domains.

Existing computational tools to predict function annotations fall into two archetypes: homology-based methods and *de novo* methods. Homology-based methods encompass the majority of traditional strategies and include sequence alignment-based tools such as Basic Local Alignment Search Tool (BLAST) (Altschul *et al.* 1990) and family classification techniques like profile Hidden Markov Models (Krogh *et al.* 1994, Eddy 2011). While effective and explainable, these methods are difficult to scale (Sanderson *et al.* 2023) and tend to underperform in remote homology situations, due to their reliance on database search via sequence similarity. On the other hand, *de novo* methods, most commonly machine-learning models, do not directly rely on the query sequence’s homology and instead construct a representation of the protein from which to infer function. Notable recent *de novo* approaches include ProteInfer (Sanderson *et al.* 2023) and DeepGOPlus (Kulmanov and Hoehndorf 2021), both of which predict Gene Ontology (GO) (Ashburner *et al.* 2000, Gene Ontology Consortium *et al.* 2023) annotations and Enzyme Commission (EC) numbers (Bairoch 2000); methods that specialize in EC number prediction, such as CLEAN and HiFi-NN (Ayres *et al.* 2025, Yu *et al.* 2023); ProtENN (Bileschi *et al.* 2022), which infers protein families from the Pfam database (Mistry *et al.* 2021); and ProteinBERT (Brandes *et al.* 2022), a GO-annotation-aware protein language model that can be fine-tuned on downstream tasks. To further improve performance, other models incorporate structural information (Kipf and Welling 2017, Gligorijević *et al.* 2021), exploit the GO hierarchy (Zhang *et al.* 2022, Sanderson *et al.* 2023), or combine deep-learning models with traditional similarity-based approaches (Bileschi *et al.* 2022, Sanderson *et al.* 2023, Shaw *et al.* 2024).

Both homology-based and existing *de novo* methods, however, suffer from meaningful drawbacks. First, both classes of methods can only predict the static set of functions in their database training set. With an average of 298 GO terms introduced and 591 GO terms deprecated each year (Supplementary Fig. S1), models become outdated almost immediately. Moreover, both approaches overlook the textual descriptions accompanying the labels, a rich source of information that may improve performance, especially on rare functions. While models like ProteinCLIP (Wu *et al.* 2024) leverage sequence-text descriptions and contrastive learning to enhance applications such as prediction of protein–protein interactions and homology detection, they are not yet tested for any type of function prediction. To bridge these gaps, recent methods for few- and zero-shot function prediction have been proposed (Xu and Wang 2022, Kulmanov and Hoehndorf 2022). Few-shot function prediction consists of predicting functions that are represented only by a small number of sequences in the training set, whereas zero-shot prediction aims to predict functions that are not present in the training set, including new functions that no protein has been annotated with. These methods typically enable zero-shot prediction by incorporating additional information during inference, such as label ontology relationships (Kulmanov and Hoehndorf 2022), gene network topology (Xu and Wang 2022), and textual descriptions (Xu and Wang 2022). Although these approaches improve performance on low-frequency labels, they underperform or are untested on traditional prediction with known labels, rely on auxiliary data that may not be available during inference, and are evaluated on artificial zero-shot test sets that overlap in time with the training data. To better simulate inference scenarios and avoid data leakage, an ideal zero-shot test set would consist of data collected after the training set date cutoff. Such an approach more accurately reflects how protein–function prediction models are used in practice and mirrors best practices from other studies (Radivojac *et al.* 2013).

We hypothesized that leveraging multimodal inputs—extracting knowledge from both a protein’s amino acid sequence and the text description of its function—could enable strong performance on protein–function prediction in both the supervised and zero-shot settings. We couple semantic protein embeddings with text embeddings from a Large Language Model (LLM) to train ProtNote, the first deep-learning model capable of both supervised and zero-shot protein–function prediction by learning over both amino acid sequences and free-form text information (Fig. 1B–D). ProtNote nears the performance of ProteInfer, the current state-of-the-art GO annotation prediction model, in the supervised setting and performs robustly in the zero-shot setting. We demonstrate how ProtNote captures nuanced sequence–function relationships and unlocks a range of biological use cases inaccessible to prior models.

## 2 Materials and methods

### 2.1 Predicting protein-text likelihood

To build a model capable of both supervised and zero-shot protein–function annotation, we formulate GO term prediction from protein sequence as a binary classification problem: given a protein-GO term pair, the model predicts if the protein has the presented GO term. Class imbalance, however, posed a challenge, as 80% of the labels are used only in 0.03% of the training sequences (Supplementary Fig. S2). We employ a two-fold approach to address this obstacle.

First, to accelerate learning from hard examples, we apply a focal loss with γ=2 and no α parameter. Let $\lambda_{j}$ denote GO term j and $x_{i}$ represent protein sequence i, where j∈{1,2,…,L}, i∈{1,2,…,N}, with L and N being the total number of GO terms and sequences, respectively. Let $p_{i,j}\in [0,1]$ denote the model’s estimated probability that sequence $x_{i}$ is annotated with GO term $\lambda_{j}$. The focal loss is defined as:

(1) $$FL_{i,j}=-{\left(1-p_{t}\right)}^{\gamma }\;\text{log}(p_{t})$$

 

$$p_{t}=\{\begin{array}{cc} p_{i,j} & \text{if}\;\text{protein}\;x_{i}\;\text{has}\;\text{annotation}\;\lambda_{j}\\ 1-p_{i,j} & \text{otherwise} \end{array}$$

Second, we sample protein sequences during training according to the inverse frequency of their function annotations. Let $f_{j}$ be the frequency of GO term $\lambda_{j}$ in the training set, then the sampling weight $w_{i}$ of a sequence i is:

(2) $$w_{i}=\sum_{j=1}^{L}\delta_{i,j}\frac{1}{\sqrt{f_{j}}},\;\delta_{i,j}=\{\begin{array}{cc} 1 & \text{if}\;\text{protein}\;x_{i}\;\text{has}\;\text{annotation}\;\lambda_{j}\\ 0 & \text{otherwise} \end{array}$$

We use the square root of the frequency to avoid unreasonably high weights resulting from the extreme label imbalance.

### 2.2 Datasets

ProtNote is trained on descriptions from GO annotations. These annotations capture our knowledge of three aspects of protein biology: molecular function, biological process, and cellular component. GO terms are phrases describing the molecular actions of gene products, the biological processes in which those actions occur, and the cellular locations where they are present. We train our model with the GO terms descriptions from the 1 July 2019 GO Annotations release.

Within the supervised setting, we leverage ProteInfer’s (Sanderson *et al.* 2023) random split, where 80%, 10%, and 10% of sequences were assigned to train, validation, and test, respectively. This dataset is constructed from the SwissProt (Boutet *et al.* 2007) section of the UniProt (The UniProt Consortium 2022) database, the world’s largest repository of manually curated, high-quality protein sequence and function information. We remove duplicated sequences and long sequences with more than 10 000 amino acids.

Since our method extends ProteInfer’s original weights and architecture, we refer to the GO terms it was trained on as *in-vocabulary* and all others as *out-of-vocabulary*. GO terms live in a directed acyclic graph, where any term can have multiple ancestors. However, UniProt entries only show the most specific known GO term, ignoring all ancestor terms. Using only the most specific terms during training would teach the model to predict positive for the specific label but negative for the parents. Therefore, we follow ProteInfer’s annotation scheme, where *all* terms—both the most specific and the ancestors—associated with a sequence are used during training.

To evaluate zero-shot performance on unseen, new GO annotations, we create a GO zero-shot test set from the 17 June 2024 GO Annotations release and the May 2024 SwissProt (Boutet *et al.* 2007) release; this zero-shot set includes (1) only sequences that were added to SwissProt between July 2019 (the version used by ProteInfer) and May 2024 and (2) only out-of-vocabulary GO terms, i.e. functions that were first described within the GO ontology after 1 July 2019. Supplementary Fig. S3 quantifies the effect of changes in the GO graph on model performance.

To explore ProtNote’s zero-shot capabilities on an out-of-distribution task, we test its performance on the prediction of EC numbers. We directly use ProteInfer’s EC numbers test set, consisting of sequences and annotations that ProtNote was not trained on, and extract the function text descriptions from ExPASy (Gasteiger *et al.* 2003). EC numbers are composed of four levels: the first level indicates the enzyme class based on the type of reaction catalyzed; the second and third levels correspond to the enzyme subclass and sub-subclass, respectively, providing more detail on the type of molecular group, bond, or product involved; and the fourth level is the enzyme identification, representing the specific enzyme and its relationship to particular metabolites or cofactors. We create a single textual description for each EC number by concatenating the descriptions of all its levels, separated by commas. Statistics for all datasets and splits are specified in Table 1.

**Table 1.** Dataset statistics.^a^

Split	Seqs	Unique seqs	Median seq length	Max seq length	Median $\frac{\text{Labels}}{\text{Seq}}$	Max $\frac{\text{Labels}}{\text{Seq}}$	Unique Labels
GO-Rand-Train	418 015	351 429	303	35 213	43	1008	31 365
GO-Rand-Val	52 841	44 362	303	10 061	43	760	21 914
GO-Rand-Test	51 751	43 575	301	11 103	43	815	22 026
GO-Zero-Shot	63 594	56 212	303	15 639	1	14	614
GO-Zero-Shot-LN	2445	2276	430	7158	1	6	402
EC-Zero-Shot	25 892	21 870	347	8903	4	22	2534

a GO-Rand-Train, GO-Rand-Val and GO-Rand-Test splits are based on those from ProteInfer (Sanderson *et al.* 2023). Not all possible GO terms are observed in all splits, and there are about 16% duplicated sequences. The length of sequences and number of annotations have extremely skewed distributions. GO-Zero-Shot and GO-Zero-Shot-LN correspond to the datasets with unseen GO annotations using all the labels and only the leaf nodes, respectively. The GO-Zero-Shot datasets have significantly fewer labels per sequence, as they only include the new labels introduced in the 2024 GO release. EC-Zero-Shot consists of unseen sequences with EC annotations, which ProtNote was not trained on. See Section 2 for details.

### 2.3 ProtNote architecture, training, and inference

An overview of the ProtNote architecture is presented in Supplementary Fig. S4. ProtNote uses ProteInfer (Sanderson *et al.* 2023) as a protein sequence encoder and the Multilingual E5 Text Embedding model (E5) (Wang *et al.* 2024) as a text encoder; both encoders are frozen throughout training. To transform the embeddings to a more suitable space and reshape them to a common size d=1024, they are each passed through independent multi-layer perceptrons (MLP) with three hidden layers of 3d=3×1024=3072 units each and an output layer of size d=1024. These embeddings are concatenated and fed through an MLP with three hidden layers of 3d=3072 units each, followed by the output neuron. The MLPs employ ReLU activations (Agarap 2018) and have no bias parameters because batch normalization (Ioffe and Szegedy 2015) is used.

While we employ E5, there are several options for the text encoder, and ProtNote’s architecture is flexible to the choice of text (and protein sequence) encoder. We chose E5 Text embeddings over the popular biomedical-specific model BioGPT (Luo *et al.* 2022), since we empirically observed improved performance with E5 as a label encoder (Supplementary Fig. S5 and Table S1). We speculate that this could be due to E5’s high-quality sentence embeddings (Wang *et al.* 2024). To the best of our knowledge, there are no biomedical-specific models that are explicitly designed to produce sentence embeddings; models like BioGPT extract sentence embeddings by averaging token-level embeddings.

During training, ProtNote uses a key set of strategies to regularize learning, generalize to rare annotations, and avoid overfitting. First, we corrupt the protein sequences by randomly applying conservative residue substitutions using the BLOSUM62 (Henikoff and Henikoff 1992) matrix. Second, we employ focal loss (Lin *et al.* 2017) and weighted sampling to mitigate the extreme label imbalance in GO annotations. There are over 32 000 annotated functions in GO, most of them with extremely low frequency; thus, without focal loss and sequence weighted sampling, the model’s natural tendency would be to focus on the most frequent classes and predict the rest of the labels as negatives. Third, the Gene Ontology offers both short and long descriptions for each GO term; we utilize both descriptions as a form of data augmentation. During training, each time an annotation is encountered, we randomly sample one of these descriptions. In contrast, during inference, we run ProtNote twice for a given annotation—once each for each of the short and long descriptions—and ensemble the probabilities by averaging them. Sometimes, GO terms can have more than two descriptions called ‘synonyms’; because not all terms have synonyms, for simplicity, we do not use them. For EC numbers, only one description is available per annotation, so the ensemble procedure simplifies to running ProtNote once per annotation. Finally, we inject random noise into the label encoder embeddings before the initial MLP. More details about these techniques are available in the Supplementary Material.

ProtNote is trained for 46 epochs on 8 × 32 GB V100 NVIDIA GPUs using an effective batch size of 256 (32 × 8) with dynamic padding. The model trains using the Adam optimizer (Kingma and Ba 2015) with a learning rate of 0.0003, employs gradient clipping set to 1, and uses mixed precision (Micikevicius *et al.* 2018). We select the model checkpoint based on the best validation performance.

### 2.4 Baselines

In the supervised setting, we compare ProtNote against ProteInfer, ProtEx, and BLAST on ProteInfer’s test set. ProtEx is a recent approach that combines self-supervised pretraining with BLAST-powered retrieval-augmented inference, leveraging exemplars from a database to enhance performance (Shaw *et al.* 2024). However, it is important to note that the comparison to ProtEx is not directly equivalent, as ProtNote neither relies on BLAST during inference nor employs self-supervised pretraining. For additional context, we also report ProtEx’s performance without exemplars included during fine-tuning and inference—which we refer to as ProtEx (no exemplars)—to provide a more direct comparison to ProtNote’s approach. The BLAST baseline identifies the most similar sequence in the training set (i.e. the top BLAST hit) for each query sequence in the test set and then uses the labels of the top hit as the predicted labels for the query sequence.

To our knowledge, no prior model has evaluated zero-shot prediction of protein–function from textual label descriptions alone. Thus, in the zero-shot setting, we devised a novel benchmark, akin to a ‘label description similarity baseline’, to lower bound our model’s performance. First, we map each out-of-vocabulary term to the closest in-vocabulary term based on the function description’s cosine similarity using E5 or BioGPT text embeddings. Then, we directly use ProteInfer to predict likelihoods for the in-vocabulary labels and simply propagate these predictions to the new labels based on the created mapping. To illustrate the label mapping, Supplementary Table S3 shows 10 examples of zero-shot terms and their closest training set terms based on the cosine similarity of E5 embeddings.

### 2.5 Evaluation metrics

To evaluate the performance of different models, we use both macro- and micro-averaged mean Average Precision (mAP), also known as the Area Under the Precision-Recall Curve (AUPRC). The mAP metric summarizes model performance across all possible prediction thresholds, eliminating threshold tuning and providing a more robust assessment of model performance than threshold-dependent metrics such as $F_{1}$ or $F_{\max}$ scores (Radivojac *et al.* 2013). We report both mAP Macro and mAP Micro because of their different virtues, although we use mAP Macro for model selection. Macro averaging offers a performance estimate that is unbiased by the GO term distribution, ensuring that each label contributes equally to the final score regardless of its prevalence in the dataset. This is particularly important given that the distribution of GO terms across sequences is extremely skewed, with 98% of GO terms appearing for at most 1% of the sequences, while the 10 most frequent labels are present for at least 58% of the sequences (Supplementary Fig. S2). On the other hand, micro averaging is influenced by the label distribution, which can be important when the goal is to optimize for the most frequently occurring outcomes.

## 3 Results

ProtNote is a deep-learning model capable of both supervised and zero-shot protein–function prediction using only protein amino acid sequences and text-based function descriptions as input. Our primary aim was to design a model capable of zero-shot inference—that is, predicting new functions that lie outside of the training set and, ultimately, that no protein has been annotated with. To achieve this, we train ProtNote to predict the likelihood that a protein is annotated with a specific function given its amino acid sequence and function text description (Fig. 1B). We leverage ProteInfer (Sanderson *et al.* 2023) and E5 Text Embeddings (Wang *et al.* 2024) to separately encode the sequence and text, respectively, and then fuse these embeddings for prediction.

We evaluate ProtNote in both the supervised (Fig. 1C) and zero-shot (Fig. 1D) settings, benchmarking against the state-of-the-art deep-learning method ProteInfer and the gold-standard, homology-based method BLAST in the supervised setting and against custom embedding-based baselines in the zero-shot setting. For completeness, we break down the models’ performance across the three GO Ontologies (biological process, cellular component, molecular function) (Supplementary Figs S6 and S7) and the seven top-level EC classes (oxidoreductases, transferases, hydrolases, lyases, isomerases, ligases, translocases) (Supplementary Fig. S8).

### 3.1 ProtNote approaches state-of-the-art performance in supervised GO function prediction

**Figure 2.**
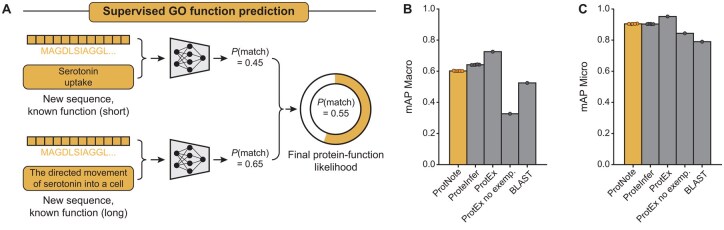
ProtNote achieves robust supervised prediction of GO function annotations. (A) In the supervised setting, ProtNote predicts protein–function likelihoods for unseen sequences with known GO annotations. ProtNote is used twice for each protein–function pair, once for the function’s short textual description and another for the long textual description. The final likelihood score is the average of the scores obtained from both descriptions. (B and C) mAP Macro (B) and mAP Micro (C) scores for GO annotation prediction in the supervised setting, comparing ProtNote (*n *=* *5 independently trained models) against ProteInfer (*n *=* *5 seeds), ProtEx with and without exemplars (*n *=* *1 seed), and BLAST (mean ±SD; numerical values and statistical tests are provided in Supplementary Table S2).

To evaluate the performance of ProtNote, we first tested the model in the supervised setting, using mAP metrics to compare our method against ProteInfer (Sanderson *et al.* 2023) and ProtEx (Shaw *et al.* 2024), two leading models for GO annotation prediction (Fig. 2A).

Overall, ProtNote nears ProteInfer’s and exceeds BLAST’s and ProtEx’s (no exemplars) performance on the test set (Fig. 2B and C) despite using a more general and scalable (Supplementary Fig. S9) architecture capable of zero-shot inference. ProteInfer retains a minor mAP Macro advantage over our model (Supplementary Fig. 2B; avg. mAP Macro of 0.6417 vs. 0.6018 for ProteInfer vs. ProtNote, respectively; two-sided Welch’s *t*-test *P* < .0001), while ProtNote is on par for mAP Micro (Fig. 2C; avg. mAP Micro of 0.9032 vs. 0.9042 for ProteInfer vs. ProtNote, respectively; two-sided Welch’s *t*-test *P* < .2298). These mAP Micro and Macro observations are consistent across the three GO Ontologies (Supplementary Fig. S6), except for the mAP Macro results for the molecular function ontology, where BLAST has a mild advantage over ProtNote (0.7292 vs. 0.7080 for BLAST vs. ProtNote, respectively).

While ProtEx achieves the highest performance across both metrics, it benefits from additional self-supervised pretraining and retrieval-augmented inference, unlike both ProtNote and ProteInfer (Fig. 2B and C). In contrast, ProtEx without exemplars—more similar in nature to ProtNote—performs significantly worse, further underscoring the value of our model’s scalable approach. This trend is also reflected in the precision-recall curves comparing the performance of ProtNote against the ProteInfer and ProtEx models (Supplementary Fig. S10).

Our experiments revealed a trade-off between supervised and zero-shot performance (Supplementary Fig. S5 and Table S1). Eliminating label embedding noise and description sampling of both short and long descriptions increased ProtNote’s performance in the supervised setting, even surpassing ProteInfer on both mAP Micro and mAP Macro metrics; however, such improvements came at the cost of zero-shot performance, resulting in greater than 5% and 4% mAP Macro decreases when ablating label embedding noise and description sampling, respectively (Supplementary Fig. S5).

### 3.2 ProtNote’s embedding space captures protein–function relationships

**Figure 3.**
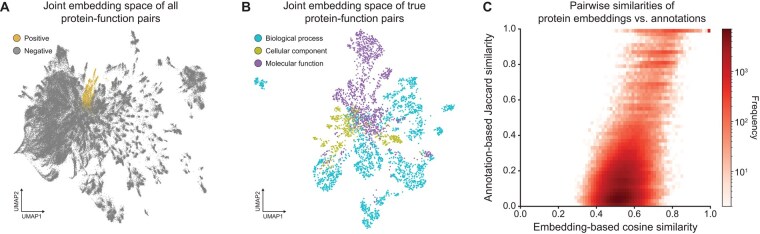
ProtNote embeddings capture protein–function relationships. All plots are based on random samples of the test set consisting of the most frequent GO annotations (*n *=* *1731). (A) UMAP projection of ProtNote output layer embeddings for protein–function pairs, where color indicates if the sequence is paired with a true positive SwissProt GO annotation or negative label (*n *=* *100 proteins × 1731 annotations = 1 731 000 pairs). (B) UMAP projection of ProtNote embeddings for the positive protein–function pairs from (A), where color indicates the GO Ontology of the pair's function (*n *=* *30 436). (C) 2D histogram of the pairwise cosine similarity between amino acid sequence embeddings and the pairwise Jaccard similarity between sequence annotations for the protein–protein pairs of the sampled test set sequences (*n *=* *800 proteins × 800 proteins = 640 000 pairs).

Motivated by the model’s performance in the supervised setting, we next inspected ProtNote’s multimodal embedding space by reducing the dimensionality using uniform manifold approximation and projection (UMAP) (Fig. 3). We observe that positive protein–function pairs (i.e. human-verified SwissProt GO annotations) cluster in a small region of the embedding space (Fig. 3A), suggesting that ProtNote learns to group semantically similar pairs closely and identifies true underlying function relationships in the protein data. Looking at the joint embeddings from only positive protein–function pairs (Fig. 3B), we observe that embeddings cluster by GO ontology groups, indicating that ProtNote’s embeddings additionally capture more nuanced biological information on function. It is worth noting that ProtNote is only trained to predict whether a pair matches and is not explicitly trained to differentiate (e.g. in a contrastive manner) among the different GO ontologies.

Based on these observations, we hypothesized that similar protein sequences would tend to have similar function labels and that ProtNote protein embeddings from the sequence projection head would reflect this relationship. To investigate this, we first define two ways to capture the similarity between a pair of proteins. First, we define the embedding-based similarity as the cosine similarity between the output embeddings from the amino acid sequence projection head. This similarity metric carries no explicit information about function annotations. Second, we specify the annotation-based similarity as the Jaccard similarity coefficient of the two proteins’ multi-hot annotation vectors. Thus, the annotation-based similarity captures no explicit sequence information. We observe a correlation between the embedding-based and annotation-based pairwise similarities (Fig. 3C; Pearson r=0.533, *P* < .0001), implying that even before the output MLP, sequence embeddings from the projection head already contain significant function information. We also observe a mild correlation between pairwise sequence and label embedding similarities (Supplementary Fig. S11; Pearson r=0.404, *P* < .0001), revealing that higher sequence similarity is associated with higher function label similarity, even though the model does not explicitly align the sequence and text embeddings.

### 3.3 ProtNote enables zero-shot function prediction

ProtNote is designed to enable zero-shot inference when given a set of unseen protein sequences and novel function descriptions. We tested ProtNote in two zero-shot tasks: predicting protein functions based on novel GO annotations and predicting EC numbers.

#### 3.3.1 Zero-shot prediction of novel GO annotations

**Figure 4.**
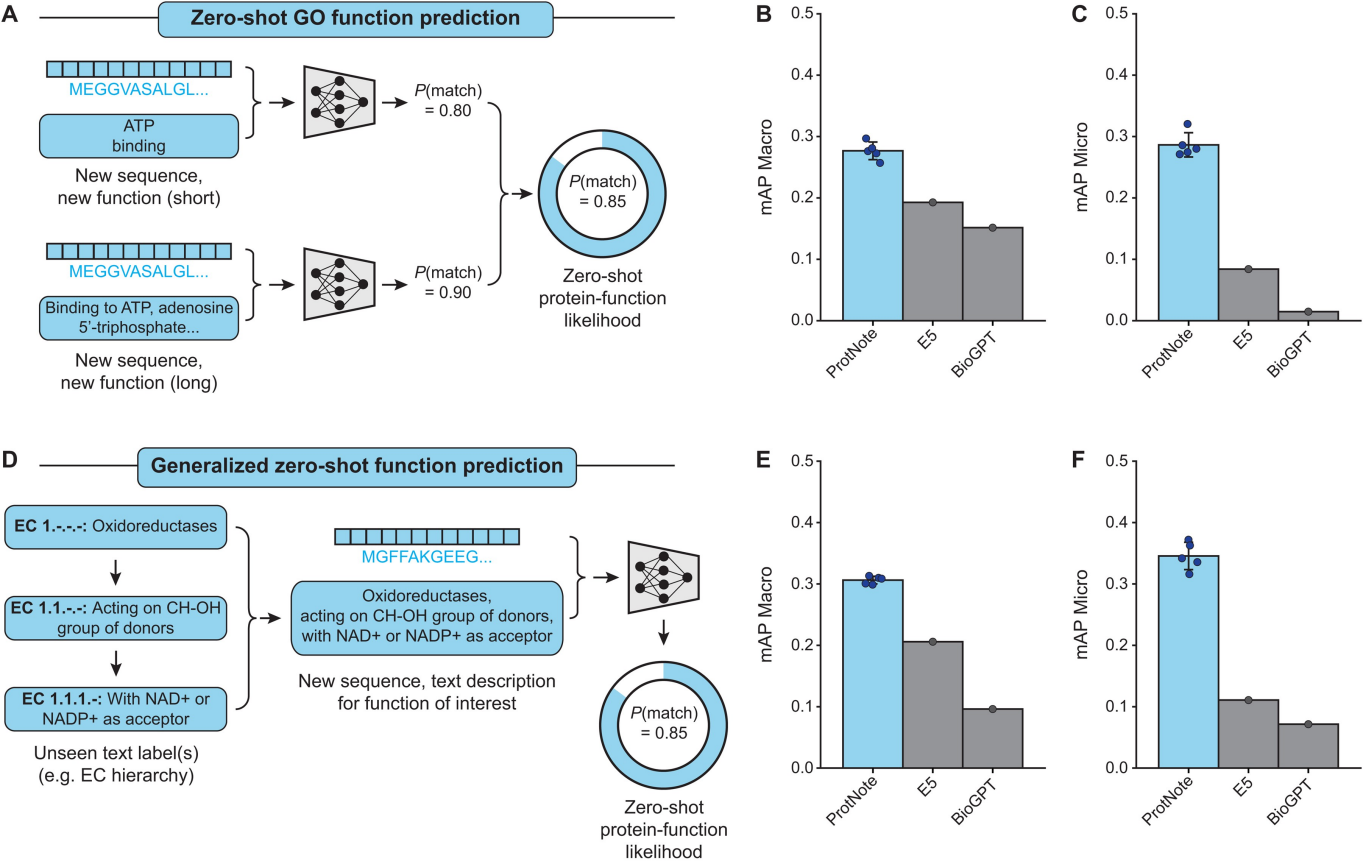
ProtNote enables generalizable zero-shot protein–function prediction. (A) In zero-shot GO function prediction, ProtNote predicts protein–function likelihoods for unseen sequences with new, unseen GO annotations. As in the supervised setting, ProtNote is used twice for each protein–function pair, and the final likelihood score is the average of the scores obtained from both short and long textual descriptions. (B and C) mAP Macro (B) and mAP Micro (C) scores for zero-shot prediction of novel GO annotations for the GO leaf nodes, comparing ProtNote (*n *=* *5 independently trained models; mean ± SD) against the Multilingual Instruct E5 and BioGPT baselines. (D) Demonstration of generalized zero-shot function prediction with Enzyme Commission (EC) numbers. A full textual description for each EC number is created by concatenating its description with those of its parents, separated by commas. A protein sequence and its corresponding aggregate text description are input into ProtNote to predict a protein–function likelihood score. (E and F) mAP Macro (E) and mAP Micro (F) scores for zero-shot prediction of EC numbers, comparing ProtNote (*n *=* *5 independently trained models; mean ± SD) against the E5 and BioGPT baselines.

To assess ProtNote’s ability to predict novel GO annotations for unseen protein sequences (Fig. 4A), we evaluated its performance in two settings: on GO annotation leaf nodes only, and on both leaf nodes *and* inferred nodes (Fig. 4B and C, Supplementary Fig. S7). Leaf nodes refer to GO terms with no children, while inferred nodes designate terms that can be derived from another annotation based on the graph hierarchy. For example, the GO terms ‘biological regulation’ (GO: 0008150) and ‘biological process’ (GO: 0065007) can be inferred from the term ‘regulation of biological process’ (GO: 0050789), as the first two are parents of the latter. When measuring zero-shot capabilities, we only consider new nodes, including both leaf and inferred nodes (Supplementary Fig. S12).

ProtNote outperforms baselines across all scenarios evaluated (Fig. 4B and C, avg. mAP Macro of 0.2139, 0.1556, 0.1236 for ProtNote, E5, BioGPT, respectively; Supplementary Fig. S7). While ProtNote exceeds the baselines on all metrics in the scenario with just leaf nodes (Fig. 4B and C), we observe the largest performance gap when comparing ProtNote’s predictions with baseline mAP Micro metrics in the scenario including both leaf and inferred nodes (Supplementary Fig. S7D), suggesting that the baselines perform poorly on frequent annotations. We note that a large portion of the performance drop between the supervised and zero-shot settings may be explained by the change in the inferred labels from changes in the GO graph (Supplementary Fig. S3). Moreover, we identify that ProtNote’s performance is superior for zero-shot terms with text descriptions similar to those seen during training (Supplementary Fig. S13A), a finding that can be used to set performance expectations in practical applications.

We also observe significant variation in performance by ontology (Supplementary Fig. S7). Namely, most models excel at predicting labels that fall within the ‘molecular function’ ontology and underperform when predicting sequence annotations from the ‘cellular component’ ontology. We hypothesize that this discrepancy could be understood from two angles: the annotations and the sequences. First, textual annotations bias toward describing molecular functions and biological processes rather than cellular components. Only 14% of the positive GO terms in the training set come from the cellular component ontology, making it the least represented ontology. Second, prior work has suggested that protein language models may learn evolutionarily conserved motifs through self-supervised pretraining (Zhang *et al.* 2024), but must be fine-tuned for prediction of subcellular location specifications (Schmirler *et al.* 2024) that may be determined by a small number of characteristic residues.

#### 3.3.2 Zero-shot prediction of EC numbers

To further assess ProtNote’s zero-shot capabilities in a markedly different task, we applied it to predicting EC numbers—a task the model was not trained on. This task involves predicting labels that describe enzyme functions using a distinct terminology that does not directly map to GO annotations (Fig. 4D). For each EC number, we constructed the input text by concatenating its description with those of its parents, separated by commas (Fig. 4D). We then used only this final text for inference without ensembling. Finally, we assessed the performance of our model across the top-level EC classes and compared it against the E5 and BioGPT baselines (Fig. 4E and F, Supplementary Fig. S8).

ProtNote outperforms the E5 and BioGPT models, both for mAP Macro (Fig. 4E; avg. scores of 0.3062, 0.2058, 0.0961 for ProtNote, E5, BioGPT, respectively) and mAP Micro (Fig. 4F; avg. scores of 0.3456, 0.1107, 0.0714 for ProtNote, E5, BioGPT, respectively). Similar to the zero-shot GO annotation task, the baselines show much lower mAP Micro than mAP Macro metrics, reflecting their poor performance on frequent EC numbers. Furthermore, ProtNote demonstrates superior performance across all seven top-level EC classes (Supplementary Fig. S8). Notably, all models perform well on ligases but struggle on translocases (Supplementary Fig. S8). Lastly, consistent with our observations for zero-shot GO annotations, ProtNote’s performance is markedly higher for zero-shot EC terms whose text descriptions are more similar to those seen during training (Supplementary Fig. S13B).

Together, these results demonstrate that ProtNote performs robustly in zero-shot prediction of protein–function annotations, as evidenced by both extrapolation to unseen GO annotations and function prediction in a completely unseen scenario of EC descriptions. Although ProtNote does not reach the metrics from the fully supervised setting, as expected, its strength in zero-shot settings highlights the effectiveness of combining amino acid sequence and text data to predict protein functions.

## 4 Discussion

In this work, we introduce ProtNote, a multimodal deep-learning model capable of supervised and zero-shot protein–function annotation. We demonstrate how ProtNote leverages unstructured text to predict the likelihood that proteins perform arbitrary functions. Importantly, we showcase that ProtNote generalizes to unseen sequences and functions by evaluating the model on newly-added GO annotations and on enzyme annotations, which were not used to train the model. We observe that this generality does not come at the cost of supervised performance; our model is also performant at predicting known GO terms, performing on par with the state-of-the-art model ProteInfer.

To the best of our knowledge, ProtNote is the first deep-learning model capable of both supervised and zero-shot protein–function prediction using only a single sequence-text pair. While related, protein naming and function prediction are distinct tasks. Sequence-to-sequence models like ProtNLM (Gane *et al.* 2025) automatically name uncharacterized proteins given their amino acid sequence, whereas ProtNote directly estimates the likelihood of a protein performing a given function. ProtNote relies neither on comparisons against thousands of other sequences, a prerequisite for homology-based methods such as BLAST (Altschul *et al.* 1990) and profile Hidden Markov Models (Krogh *et al.* 1994, Eddy 2011), nor on difficult-to-obtain ancillary information, such as ProTranslator’s protein Mashup representations and GeneCard information (Xu and Wang 2022). Instead, ProtNote depends solely on a protein’s amino acid sequence and a text description for zero-shot inference. This flexibility contrasts with recent machine-learning-based attempts that depend on multiple—and often scarce—inputs from different modalities (Xu and Wang 2022, Kulmanov and Hoehndorf 2022) and evaluate zero-shot capabilities based on synthetically crafted datasets rather than real time-based splits. Compared to traditional supervised approaches, ProtNote is more robust to changes in the GO, as it is less impacted by new, removed, or redefined terms.

Despite its demonstrated performance and versatility, ProtNote leaves significant possibilities for future improvement and expansion. We train only on the GO dataset, which inherently introduces several obstacles. First, while ProtNote is more robust to changes in the GO relative to supervised approaches trained on a fixed set of terms, like all GO-trained models, it remains prone to prediction errors driven by changes in the GO graph. As shown in Supplementary Fig. S1, changes in the graph structure directly modify the annotations implied by GO ancestry. Second, the model might overfit to text descriptions with similar content and style to those observed in the Ontology. The sequence–function matching formulation also has some drawbacks. To make predictions, ProtNote must run inference through an MLP for every protein-text pair, requiring more computational resources than other strategies. Further, ProtNote does not leverage relationships between functions to improve its predictions.

There are a number of promising research angles that could be explored to improve model performance and enable additional applications. First, training text may be expanded from GO function annotations to include other categories such as subcellular location, catalytic activity, involvement in diseases, and membership in protein families (The UniProt Consortium 2022, Bairoch 2000, Hamosh *et al*. 2000, Schomburg *et al.* 2002, Sigrist *et al.* 2012, Mistry *et al.* 2021). Second, ProtNote’s joint MLP could be removed in favor of using a contrastive learning approach with LLM fine-tuning for faster inference, while learning a single representation in a joint embedding space. Third, a more principled weighted sampling scheme could be used instead of weighting by the sum of individual GO term weights. Finally, the development and use of different text encoders could be explored. We tested general-domain sentence transformers such as E5 (Wang *et al.* 2024) and biomedical-specific masked language models that are not trained to produce sentence embeddings (Gu *et al.* 2022, Luo *et al.* 2022), and observed that general-domain sentence transformers yielded improved performance. Future work to train and apply biomedical-specific sentence transformers, which are currently rare, will help delineate whether biomedical-specific training provides benefits relative to general language models.

In sum, ProtNote provides a multimodal framework for protein–function prediction and generalizes to functions beyond those on which it was trained. Our work is an important step toward general-purpose function prediction models that are robust to the dynamic, ever-evolving landscape of proteins.

## Supplementary Material

btaf170_Supplementary_Data

## Data Availability

Code is available at https://github.com/microsoft/protnote. Model weights, datasets, and computed metrics are available at https://zenodo.org/records/13897920.
