# Supplementary material for: ProtNote: a multimodal method for protein–function annotation
Source: Bioinformatics. 2025 Apr 15;41(5):btaf170. doi: 10.1093/bioinformatics/btaf170 (PMC12054973; doi:10.1093/bioinformatics/btaf170)
Supplement: btaf170_Supplementary_Data [file btaf170_supplementary_data.zip › ProtNote S.I.pdf]

SUPPLEMENTARY INFORMATION FOR:

ProtNote: a multimodal method for protein-function annotation

Samir Char[1], Nathaniel Corley[2,§], Sarah Alamdari[3], Kevin K. Yang[3], and Ava P. Amini[3,†]

[1]Microsoft Cloud & AI, Microsoft, Redmond, WA, USA 98052
[2]Microsoft Cloud & AI, Health & Life Sciences, Microsoft, Redmond, WA, USA 98052
[3]Microsoft Research, Microsoft, Cambridge, MA, USA 02142
[†]Corresponding author. ava.amini@microsoft.com
[§]Current affiliation: Institute for Protein Design, University of Washington, Seattle, WA, USA 98195

A. Variability in the Gene Ontology (GO) and its impact on model performance

The GO is far from stable. A key aspect of the GO is that the GO Consortium is constantly refining it for the benefit of science. Despite the clear advantages of this ongoing curation, a major drawback is that the ontology changes frequently across releases, potentially affecting all downstream analyses that rely on it. This problem is of such significance that researchers have discussed it extensively and developed tools to identify and track changes in the GO [1]. There are three major sources of change in the GO that impact supervised models in different ways: GO Terms can be added or removed (Fig. S1A), their definitions may change (Fig. S1B), and their locations in the graph can evolve (Fig. S1C). Without retraining, supervised models are unable to predict new terms, while they will continue to predict old terms, introducing false positives. When a term's definition changes, the predictions of a supervised model will have inconsistent interpretations. Furthermore, a change in the GO graph may modify the inferred labels of a given term. Although the third problem (Fig. S1C) impacts all models, the first two (Fig. S1A-B) do not alter ProtNote, since it is able to predict likelihoods for new or changed terms.

Since changes in the GO graph influence the performance of all models, we developed an evaluation to isolate and quantify this effect. We created a new test set with the same protein sequences as ProteInfer's 2019 test set, but with updated annotations based on the May 2024 GO release. Given that the latest release contains new terms, we only kept the terms that were seen during ProteInfer's training in its original dataset. ProteInfer's mAP Macro and Micro decreased by 11.67 and 22.51% (absolute), respectively, on the updated test set with the same sequences but updated annotations (Fig. S3). These results demonstrate that supervised models degrade quickly for this task, highlighting the need for more flexible alternatives, and underscore the difficulty of zero-shot inference for this task.

Finally, we note that the distribution of GO terms is highly imbalanced – most of the labels are used rarely, i.e. for a small proportion of protein sequences, while the top labels in the GO are used very frequently (Fig. S2).

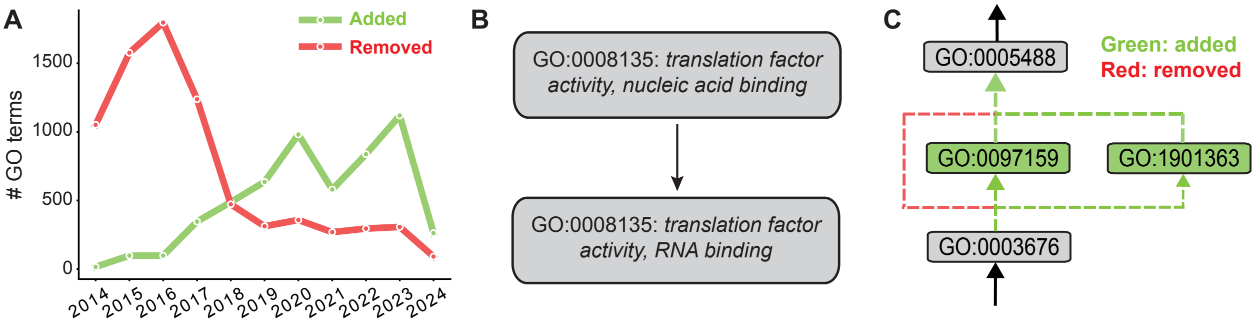

Fig. S1. Changes in the GO are frequent and diverse. (A) Addition and removal of GO terms over time for the last 10 years. **(B)** Example of a GO term for which the definition changed from one release to another. **(C)** Example of a simple structure change for the parent nodes of GO term GO:0003676.

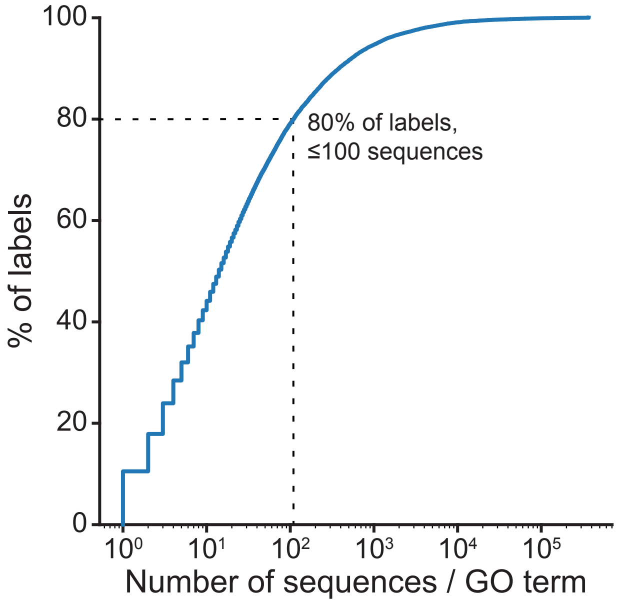

Fig. S2. Cumulative distribution function of the number of protein sequences per GO term. The x-axis represents the number of protein sequences associated with each GO term, while the y-axis shows the cumulative proportion of GO terms used for a given number of sequences. Most of the labels are used infrequently, while the top labels are used extensively.

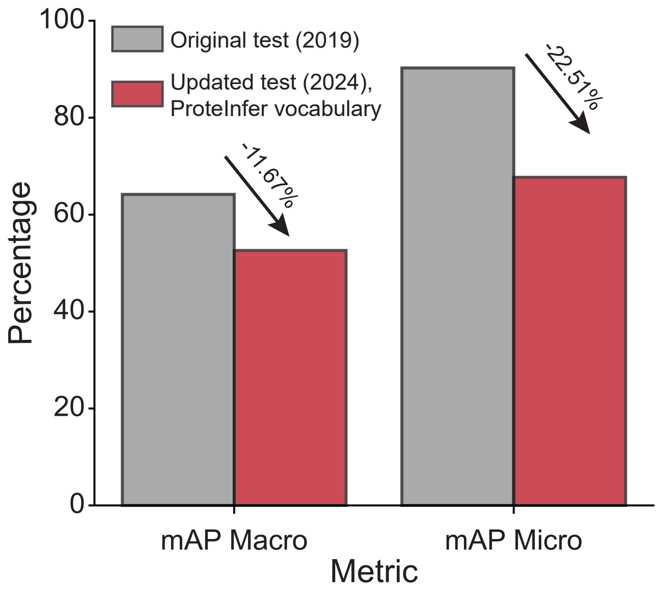

Fig. S3. Impact of changes in the GO graph on ProteInfer baseline performance. mAP Macro and mAP Micro performance of the ProteInfer baseline on the original 2019 test set (grey) and on an updated test set (red) with the same protein sequences but with applicable GO terms based on the May 2024 GO release.

B. Detailed results

A detailed schematic of the ProtNote modeling framework and architecture is provided in Figure S4. We perform a series of ablations to assess the impact of different design choices on ProtNote's performance in both the supervised and zero-shot settings (Fig. S5). To thoroughly evaluate model performance, we assess ProtNote's predictions across the three GO Ontologies for the supervised (Fig. S6) and zero-shot (Fig. S7) settings, and the seven top-level EC numbers (Fig. S8) in the zero-shot setting.

In all settings, we report two metrics: the macro-averaged mean Average Precision (mAP macro) and the micro-averaged mean average precision (mAP micro) scores. However, in our experiments, we optimize for mAP Macro.

C. Evaluation metrics

The macro-averaged mAP is calculated by first determining the Average Precision (AP) – synonymous with the Area Under the Precision-Recall Curve (AUPRC) – for each class (i.e., functional annotation) separately, and then taking the arithmetic mean across the Average Precision values of all classes. Since this approach assigns equal weight to each class, macro-averaging is unaffected by class frequency. In contrast, micro-averaging disregards the distinction between classes and treats the problem as a binary classification: "correct" versus "incorrect" predictions. Consequently, the AUPRC calculated using this method is biased towards the dominant classes, as it aggregates the total true positives, false positives, and false negatives.

To compute the mAP micro and mAP macro, we use TorchEval's [3, 4] BinaryAUPRC and MultilabelAUPRC, respectively. In both cases, the library calculates the area under the curve using the Riemann integral (i.e., the "rectangle rule").

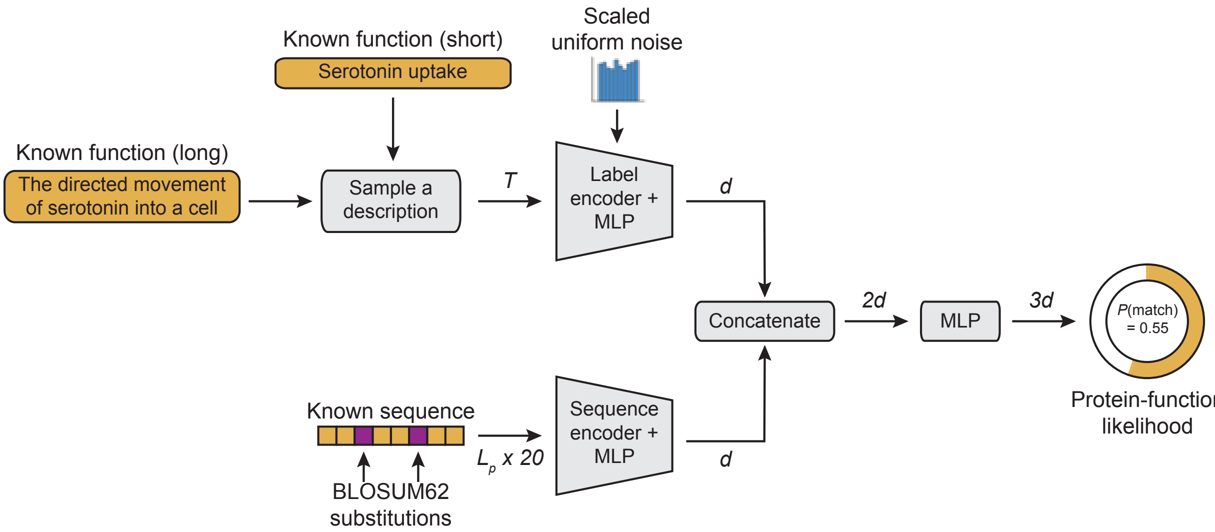

Fig. S4. ProtNote modeling framework. During training, a protein sequence-function annotation pair is sampled. As augmentation, the protein sequence is corrupted using BLOSUM62 substitutions, and then a free-text function annotation is sampled, as either the long or short description for that function. Subsequently, the text and sequence are embedded using Multilingual E5 Text embeddings and ProteInfer, respectively, and then each embedding is projected, using a multi-layer perceptron (MLP), to a new space with common dimensionality $d = 1024$. To improve generalization noise is added to the label embeddings before the projection head. Finally, the label and sequence embeddings are concatenated and pass through a final MLP to output a protein-function likelihood.

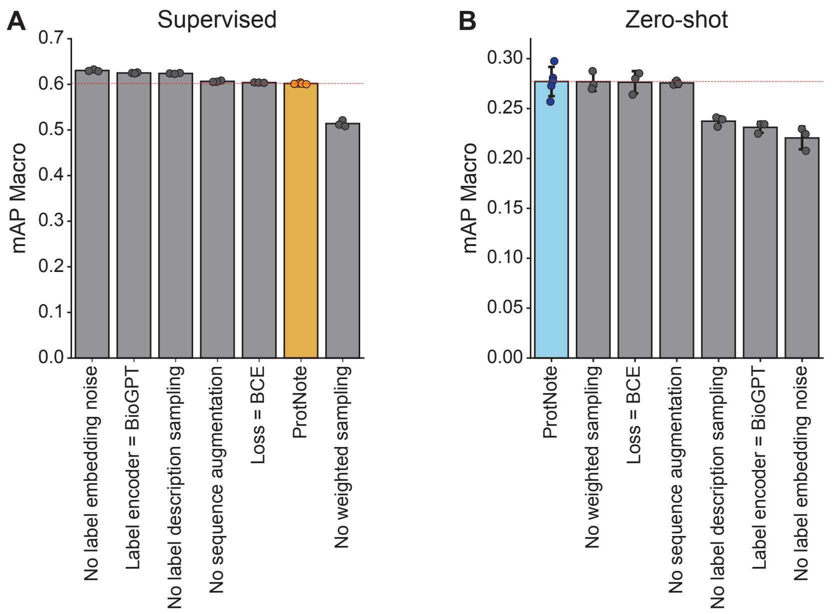

Fig. S5. Supervised and zero-shot performance of ablated models. (A) mAP Macro scores for GO annotation prediction in the supervised setting, for ProtNote (yellow; *n*=5 independently trained models) and model ablations across different design choices (grey; *n*=3 independent models). **(B)** mAP Macro scores for zero-shot prediction of novel annotations for GO leaf nodes for ProtNote (yellow; *n*=5 independent models) and model ablations across different design choices (grey; *n*=3 independent models).

Table S1. Table for Fig S5: Supervised and zero-shot performance of ablated ProtNote models. mAP Macro mean and standard deviation (s.d) for GO annotation prediction in the supervised (left) and zero-shot (right) setting for ProtNote (*n*=5 independent models) and ablated variants (*n*=3 independent models for each variant). Numbers in bold represent the best model variant for each setting.

	Supervised		Zero-shot	
Model	Mean	s.d	Mean	s.d
No sequence augmentation	0.6065	0.0013	0.2757	0.0021
No label description sampling	0.6239	0.0007	0.2374	0.0049
No label embedding noise	**0.6304**	0.0016	0.2205	0.0114
Label encoder = BioGPT	0.6250	0.0009	0.2312	0.0054
Loss = BCE	0.6038	0.0003	0.2764	0.0111
No weighted sampling	0.5140	0.0052	0.2770	0.0094
ProtNote	0.6019	0.0011	**0.2771**	0.0146

Table S2. Detailed performance metrics for supervised prediction of GO function annotations over the three GO ontologies. mAP Macro and mAP Micro average scores (± s.d) for GO annotation prediction in the supervised setting, split by the three ontologies of the GO, Biological Process (BP), Cellular Component (CC), and Molecular Function (MF), with "All" showing the overall performance across all ontologies. ProtNote and ProteInfer metrics computed over 5 independently trained models with different random seeds, while BLAST and ProtEx metrics are computed with a single model and therefore have no s.d. Bold numbers represent the best model for each scenario and metric. Asterisks indicate significance levels as follows: * for $p < 0.05$, ** for $p < 0.01$, *** for $p < 0.001$, and **** for $p < 0.0001$. Significance between ProtNote and ProteInfer was determined using Welch's t-test (n = 5 random seeds each models), while significance between ProtNote and the other models was computed using a two-sided t-test (n = 5 for ProtNote, n = 1 for other).

Ontology	Model	mAP Macro	mAP Micro
All	ProtEx	**72.56******	**95.17******
	ProtEx (no exemplars)	32.68****	84.36****
	ProtNote	60.19 (± 0.11)	90.42 (± 0.14)
	ProteInfer	64.18**** (± 0.15)	90.32 (± 0.08)
	Blast	52.49****	78.99****
BP	ProtEx	**67.38******	**93.40******
	ProtEx (no exemplars)	28.28****	79.77****
	ProtNote	56.87 (± 0.12)	87.86 (± 0.24)
	ProteInfer	58.77**** (± 0.23)	87.43* (± 0.12)
	Blast	45.41****	74.45****
CC	ProtEx	**74.18******	**93.84******
	ProtEx (no exemplars)	28.86****	82.05****
	ProtNote	58.72 (± 0.34)	89.69 (± 0.12)
	ProteInfer	63.75**** (± 0.22)	89.22*** (± 0.06)
	Blast	54.38****	76.34****
MF	ProtEx	**87.45******	**98.92******
	ProtEx (no exemplars)	47.50****	94.13****
	ProtNote	70.77 (± 0.13)	96.60 (± 0.04)
	ProteInfer	80.59**** (± 0.16)	96.45** (± 0.06)
	Blast	72.92****	91.24****

Table S3. Examples of zero-shot annotations. Examples of zero-shot test terms along with their closest training set term based on cosine similarity between text descriptions using the E5 embeddings.

Test Term	Train Term	Test Term Description	Train Term Description
EC:1.3.1.22	GO:0047751	a 3-oxo-5alpha-steroid + NADP(+) = a 3-oxo-Delta(4)-steroid + H(+) +NADPH.	3-oxo-5alpha-steroid 4-dehydrogenase (NADP+) activity
EC:1.1.1.44	GO:0047100	6-phospho-D-gluconate + NADP(+) = CO2 + D-ribulose 5-phosphate + NADPH.	glyceraldehyde-3-phosphate dehydrogenase (NADP+) (phosphorylating) activity
EC:2.4.1.12	GO:0102751	[(1->4)-beta-D-glucosyl](n) + UDP-alpha-D-glucose = [(1->4)-beta-D-glucosyl](n+1) + H(+) + UDP.	UDP-alpha-D-glucose:glucosyl-glycogenin alpha-D-glucosyltransferase activity
EC:2.4.2.8	GO:0008893	diphosphate + IMP = 5-phospho-alpha-D-ribose 1-diphosphate +hypoxanthine.	guanosine-3′,5′-bis(diphosphate) 3′-diphosphatase activity
EC:2.4.1.267	GO:0033185	a dolichyl beta-D-glucosyl phosphate + alpha-D-Man-(1->2)-alpha-D-Man-(1->2)-alpha-D-Man-(1->3)-[alpha-D-Man-(1->2)-alpha-D-Man-(1->3)-[alpha-D-Man-(1->2)-alpha-D-Man-(1->6)]-alpha-D-Man-(1->6)]-beta-D-Man-(1->4)-beta-D-GlcNAc-(1->4)-alpha-D-GlcNAc-diphosphodolichol = a dolichylphosphate + alpha-D-Glc-(1->3)-alpha-D-Man-(1->2)-alpha-D-Man-(1->2)-alpha-D-Man-(1->3)-[alpha-D-Man-(1->2)-alpha-D-Man-(1->3)-[alpha-D-Man-(1->2)-alpha-D-Man-(1->6)]-alpha-D-Man-(1->6)]-beta-D-Man-(1->4)-beta-D-GlcNAc-(1->4)-alpha-D-GlcNAc-diphosphodolichol + H(+).	dolichol-phosphate-mannose synthase complex
GO:0106244	GO:0009905	eupatolide synthase activity	ent-copalyl diphosphate synthase activity
GO:0140627	GO:0071596	ubiquitin-dependent protein catabolic process via the C-end degron rule pathway	ubiquitin-dependent protein catabolic process via the N-end rule pathway
EC:4.3.1.24	GO:0006558	L-phenylalanine = (E)-cinnamate + NH4(+).	L-phenylalanine metabolic process
GO:0160143	GO:0120159	21S rRNA pseudouridine(2819) synthase activity	rRNA pseudouridine synthase activity
GO:0140439	GO:0019707	protein-cysteine S-stearoyltransferase activity	protein-cysteine S-acyltransferase activity

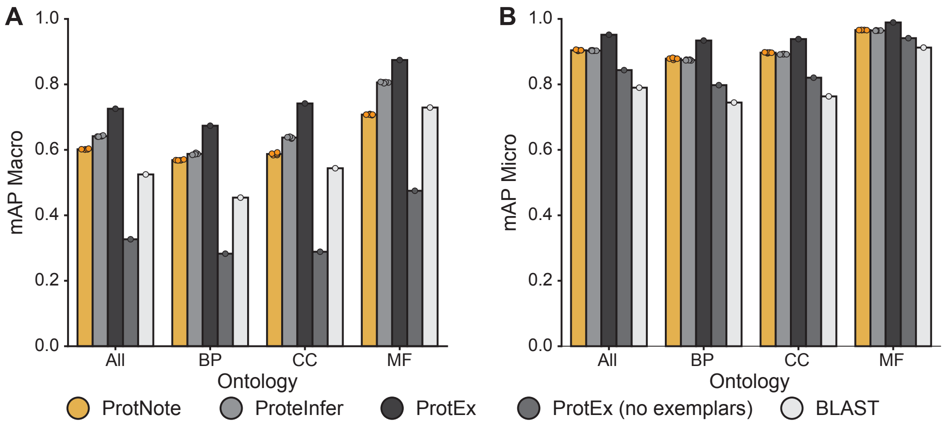

Fig. S6. Detailed performance metrics for supervised prediction of GO function annotations over the three GO ontologies. (A-B) mAP Macro (A, left) and mAP Micro (B, right) scores for GO annotation prediction in the supervised setting, split by the three ontologies of the GO, Biological Process (BP), Cellular Component (CC), and Molecular Function (MF), with "All" showing the overall performance across all ontologies. ProtNote (yellow; *n*=5 independently trained models) is compared against ProteInfer (gray; *n*=5 seeds), black; ProtEx (*n*=1 seed), ProtEx (dark gray; no exemplars) (*n*=1 seed), and BLAST (mean ± s.d.).

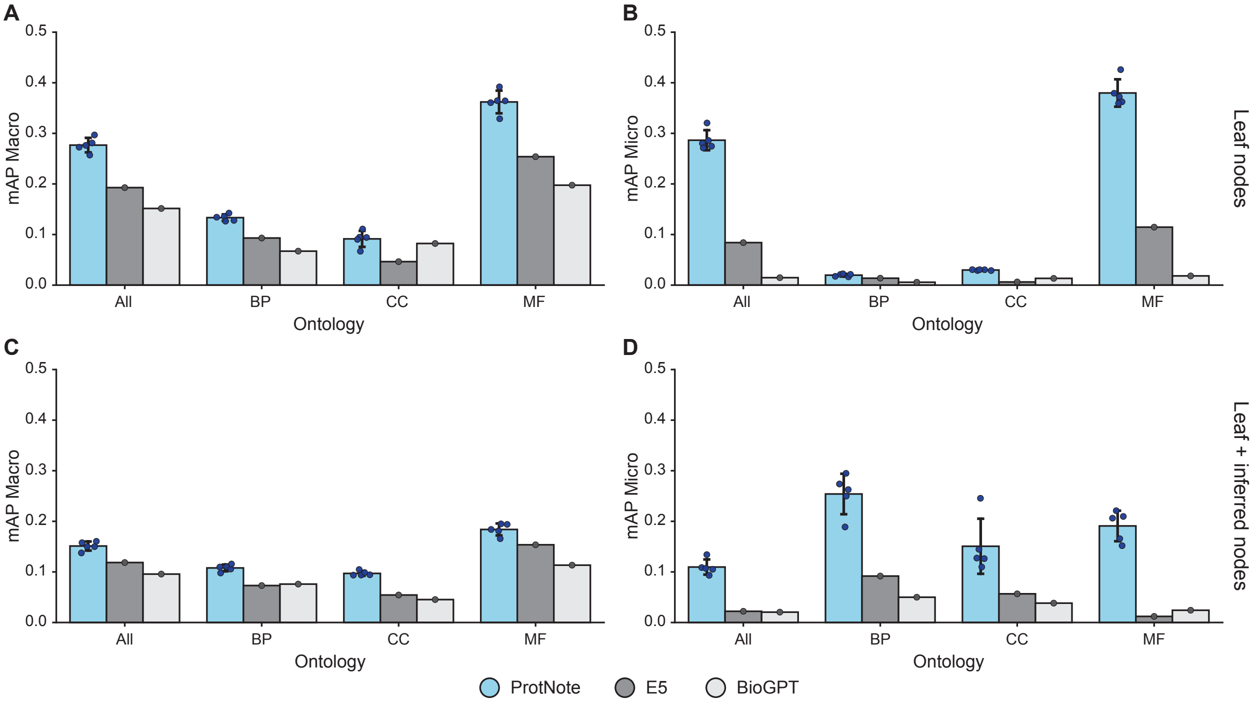

Fig. S7. Detailed performance metrics for zero-shot prediction of novel, unseen GO function annotations over the three GO ontologies. **(A-D)** mAP Macro (left column) and mAP Micro (right column) scores for zero-shot prediction of novel GO annotations, split by the three ontologies of the GO, Biological Process (BP), Cellular Component (CC) and Molecular Function (MF), with "All" showing the overall performance across all ontologies. **(A-B)** show performance only for the GO leaf nodes; **(C-D)** show performance across all nodes: leaf nodes plus those inferred from the GO graphs. In the zero-shot setting, ProtNote (blue; *n*=5 independently trained models) is compared against the label similarity baseline using E5 (grey) and BioGPT (white) for label embedding (mean ± s.d.).

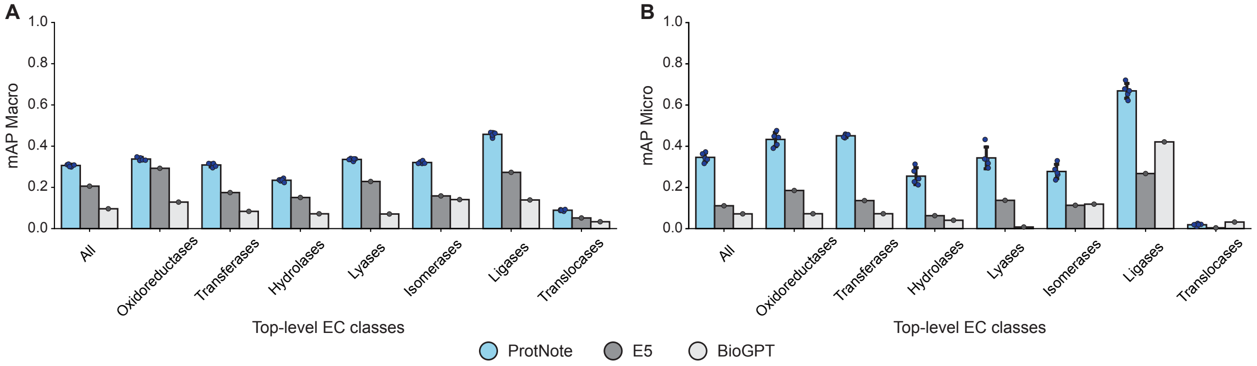

Fig. S8. Detailed performance metrics for zero-shot prediction of Enzyme Commission (EC) numbers over the top-level enzyme classes. (A-B) mAP Macro (A, left) and mAP Micro (B, right) scores for zero-shot prediction of EC Numbers, split by the seven top-level EC numbers. ProtNote (blue, n=5 independently trained models) is compared against the label similarity baseline using E5 (grey) and BioGPT (white) for label embedding (mean $\pm$ s.d.).

D. Augmentation and regularization

We employ two data augmentation techniques and one regularization strategy to improve ProtNote's generalization capacity.

Sequence residue substitution. To increase the robustness of ProtNote, we implement a data augmentation pipeline for all sequences, where each residue in a sequence has a probability p of being replaced with an amino acid sampled from the BLOSUM62 matrix, and a $1 - p$ of being left unchanged. To sample from the BLOSUM62 matrix, we select the conservative substitutions for the given amino acid, convert the substitution scores into probabilities, and then sample a substitution based on this probability distribution. The probability p is treated as a hyperparameter and set to 0.1 in our experiments.

Label description sampling. Terms in the GO database contain two attributes known as "name" and "definition". Names are typically a one sentence description of the function, while definitions are usually a longer paragraph. Definitions are richer but often contain redundant or unnecessary information, while names have limited content but are less noisy. During our initial studies we found that using names instead of definitions resulted in better performance. However, to leverage both sources of information, we trained with both description types by randomly sampling the name or the definition of any GO term with equal probability. For inference on the validation and test sets, the model makes two predictions for each protein-function pair, one using names and the other using labels, and we ensemble these via averaging. In the main text and Fig. S4, these are referenced as the "short" and "long" descriptions corresponding to the GO "name" and "definition" attributes, respectively.

Label embedding noising. Inspired by the success of introducing embedding noise during LLM fine-tuning [2] as a regularization technique, we add to the label embeddings a sample of uniform random noise in the range $[-1, 1]$, scaled by a factor of $\alpha / \sqrt{d}$. The scalar α is a hyperparameter and set to 20 in our experiments, and d is the dimensionality of the embedding space, set to 1024.

E. Additional figures

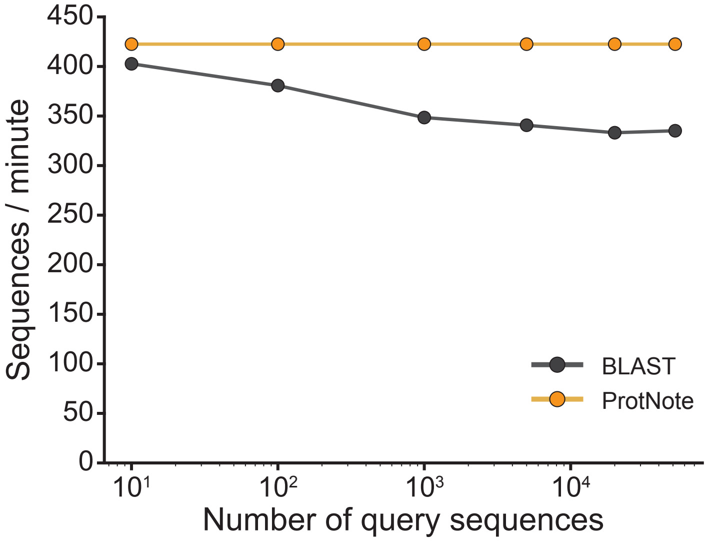

Fig. S9. ProtNote is more efficient than BLAST at protein function prediction. Number of query sequences processed per minute (y-axis) for query sets of different sizes (x-axis) for ProtNote (yellow) vs BLAST (grey). ProtNote's efficiency is constant as the query set increases, whereas BLAST's efficiency decreases as the query set increases. Note that BLAST efficiency depends additionally on the training set size, which for these experiments was held constant.

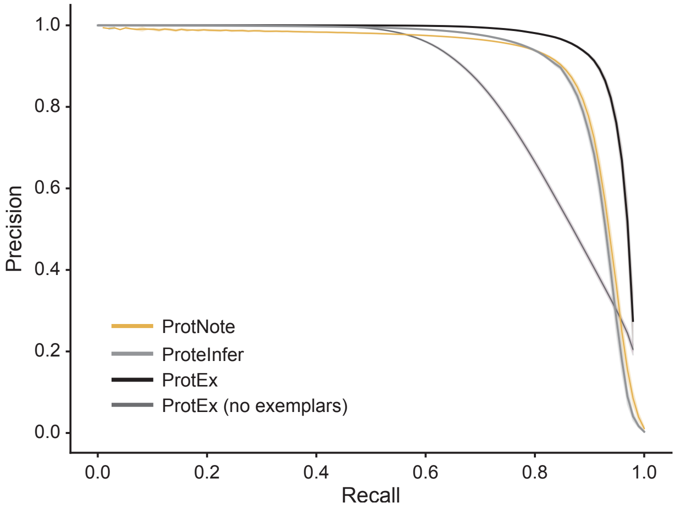

Fig. S10. Precision-Recall (PR) curves for supervised prediction of GO function annotations. Micro-averaged PR curve for ProtNote (yellow; $n = 5$ independent models; mean $\pm$ s.d), ProteInfer (grey; $n = 5$ independent models; mean $\pm$ s.d), ProtEx (black; $n = 1$ model), and ProtEx (no exemplars) (dark grey; n=1 model). BLAST is excluded as it lacks output probabilities necessary for PR curve calculation.

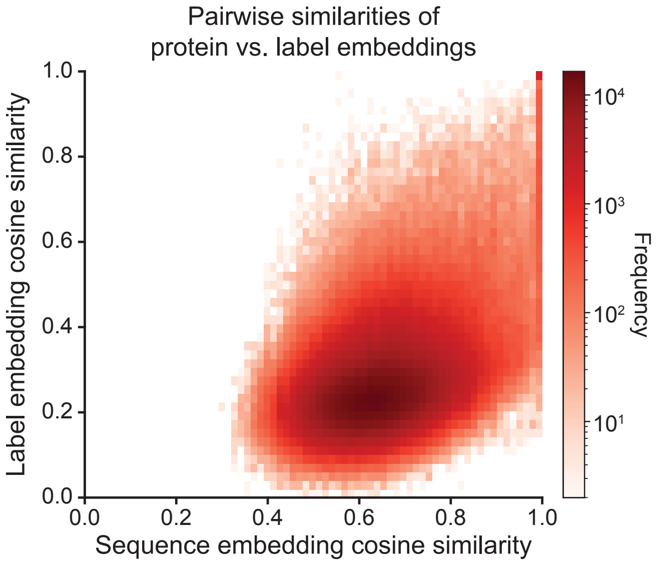

Fig. S11. Correlation between pairwise similarities for protein sequence and label embeddings. All plots are based on a subset of the test set consisting of the most frequent GO annotations (n=1,502) for a random sample of sequences (n=800). Each label is represented in two ways: first, by the median sequence embedding of the sequences annotated with that label; second, with the label embedding from the label encoder projection head (before the final MLP). The plot shows a 2D histogram of the pairwise cosine similarities between the median sequence embeddings of the positive annotations (x-axis) versus the pairwise cosine similarities of the label embeddings (y-axis) for the label-label pairs of the sampled test set sequences (n=1502 median sequence embeddings x 1502 label embeddings = 2,256,004 pairs).

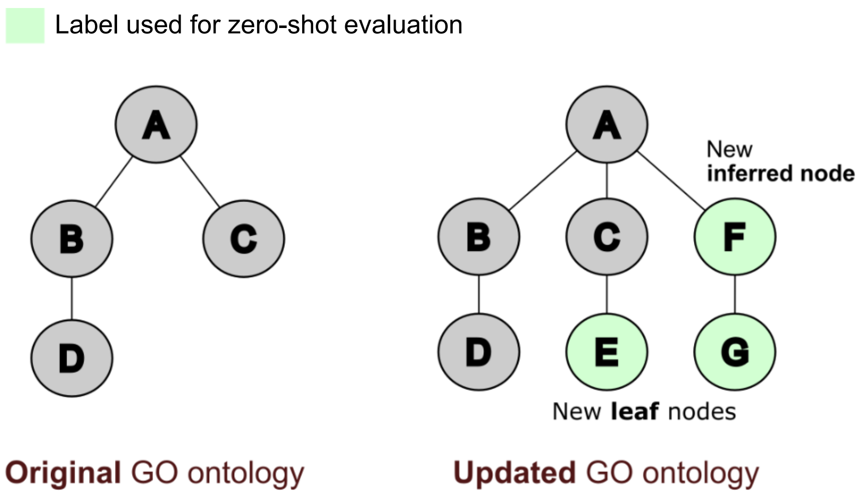

Fig. S12. Labels used to assess zero-shot performance. Zero-shot capabilities are assessed using only new labels, from both leaf and inferred nodes. Leaf nodes refer to GO terms with no children, while inferred nodes designate terms that can be derived from another annotation based on the graph hierarchy.

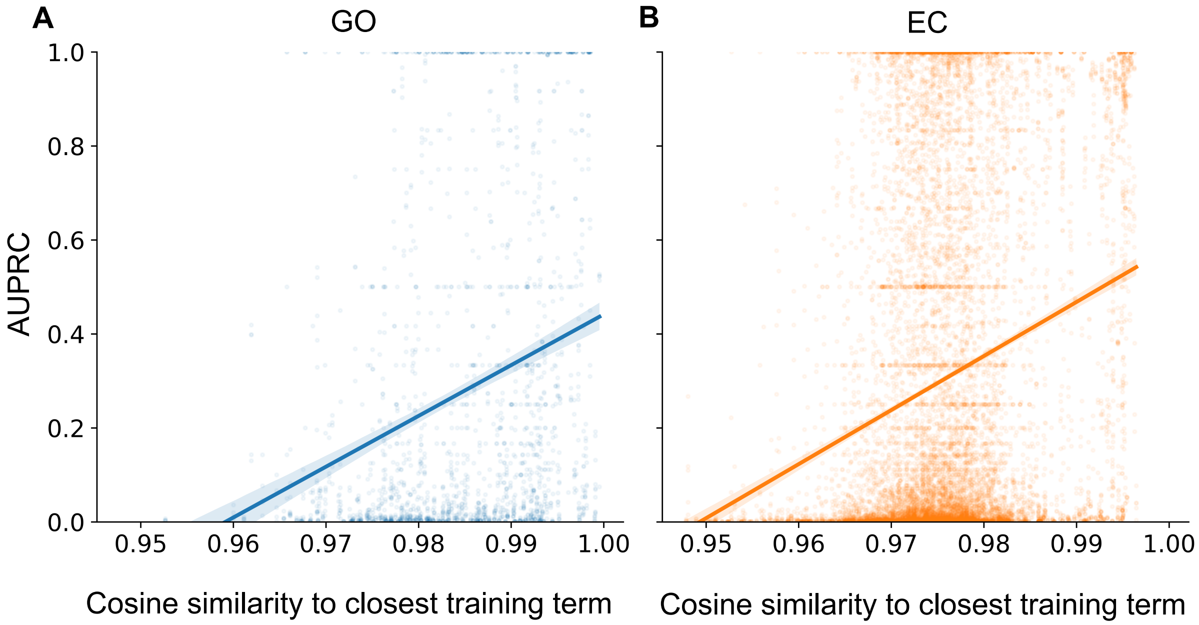

Fig. S13. ProtNote's zero-shot performance is higher for new annotations that are more similar to those seen during training. For each zero-shot task, *out-of-vocabulary* terms are mapped to the closet *in-vocabulary* term based on the cosine similarity of the text descriptions using E5 embeddings. The scatter plot shows the Area Under the Precision-Recall Curve (AUPRC; y-axis) for each *out-of-vocabulary* term (points) as a function of the cosine similarity to its closest *in-vocabulary* term (x-axis). The lines represent linear regressions with 95% confidence intervals. **A** and **B** show the plots for GO (blue) and EC (orange) zero-shot tasks, respectively.

REFERENCES

1. Matthew "Jacobson, Adriana Estela Sedeño-Cortés, and Paul" Pavlidis. "monitoring changes in the gene ontology and their impact on genomic data analysis". *Gigascience*, 7(8), August 2018.
2. Neel Jain, Ping-yeh Chiang, Yuxin Wen, John Kirchenbauer, Hong-Min Chu, Gowthami Somepalli, Brian R. Bartoldson, Bhavya Kailkhura, Avi Schwarzschild, Aniruddha Saha, Micah Goldblum, Jonas Geiping, and Tom Goldstein. Neftune: Noisy embeddings improve instruction finetuning. *CoRR*, abs/2310.05914, 2023.
3. Adam Paszke, Sam Gross, Francisco Massa, Adam Lerer, James Bradbury, Gregory Chanan, Trevor Killeen, Zeming Lin, Natalia Gimelshein, Luca Antiga, Alban Desmaison, Andreas Kopf, Edward Yang, Zachary DeVito, Martin Raison, Alykhan Tejani, Sasank Chilamkurthy, Benoit Steiner, Lu Fang, Junjie Bai, and Soumith Chintala. Pytorch: An imperative style, high-performance deep learning library. In H. Wallach, H. Larochelle, A. Beygelzimer, F. d'Alché-Buc, E. Fox, and R. Garnett, editors, *Advances in Neural Information Processing Systems*, volume 32. Curran Associates, Inc., 2019.
4. Adam Paszke, Sam Gross, Francisco Massa, Adam Lerer, James Bradbury, Gregory Chanan, Trevor Killeen, Zeming Lin, Natalia Gimelshein, Luca Antiga, Alban Desmaison, Andreas Kopf, Edward Yang, Zachary DeVito, Martin Raison, Alykhan Tejani, Sasank Chilamkurthy, Benoit Steiner, Lu Fang, Junjie Bai, and Soumith Chintala. Torcheval: A library for model evaluation, 01 2023.
